# Serotonin Receptor 2B Mediates Mechanical Hyperalgesia by Regulating Transient Receptor Potential Vanilloid 1

**DOI:** 10.1007/s12031-015-0693-4

**Published:** 2015-12-03

**Authors:** Yeu-Shiuan Su, Yuan-Yi Chiu, Shih-Yuan Lin, Chih-Cheng Chen, Wei-Hsin Sun

**Affiliations:** Department of Life Sciences, National Central University, Jhongda Road 300, Jhongli, 32054 Taiwan Republic of China; Institute of Biomedical Sciences, Academia Sinica, Taipei, Taiwan Republic of China; Institute of Systems Biology and Bioinformatics, National Central University, Jhongli, Taiwan Republic of China; Center for Biotechnology and Biomedical Engineering, National Central University, Jhongli, Taiwan Republic of China

**Keywords:** Serotonin, 5-HT_2B_, Mechanical hyperalgesia, Protein kinase Cε, Transient receptor potential vanilloid 1

## Abstract

Serotonin [5-hydroxytryptamine (5-HT)], an inflammatory mediator, contributes to inflammatory pain. The presence of multiple 5-HT subtype receptors on peripheral and central nociceptors complicates the role of 5-HT in pain. Previously, we found that 5-HT_2B/2C_ antagonist could block 5-HT-induced mechanical hyperalgesia. However, the types of neurons or circuits underlying this effect remained unsolved. Here, we demonstrate that the G_q/11_-phospholipase Cβ-protein kinase C_ε_ (PKCε) pathway mediated by 5-HT_2B_ is involved in 5-HT-induced mechanical hyperalgesia in mice. Administration of a transient receptor potential vanilloid 1 (TRPV1) antagonist inhibited the 5-HT-induced mechanical hyperalgesia. 5-HT injection enhanced 5-HT- and capsaicin-evoked calcium signals specifically in isolectin B_4_ (IB_4_)-negative neurons; signals were inhibited by a 5-HT_2B/2C_ antagonist and PKCε blocker. Thus, 5-HT_2B_ mediates 5-HT-induced mechanical hyperalgesia by regulating TRPV1 function.

## Introduction

Serotonin [5-hydroxytryptamine (5-HT)], released from platelets, mast cells, and endothelial cells into the inflamed site, is an important inflammatory mediator causing pain and hyperalgesia (Dray 1995; Sommer 2004). More than one subtype of serotonin receptor is present in peripheral nociceptive afferents (Hoyer et al. 2002). so each receptor may have distinct functions in 5-HT-induced pain. Use of specific receptor agonists, antagonists, or gene-targeting techniques have clarified the roles of 5-HT-receptor subtypes in pain. 5-HT-induced mechanical hyperalgesia is inhibited by 5-HT_2B/2C_ antagonist but not 5-HT_1A_, 5-HT_2A_, or 5-HT_3_ antagonist (Lin et al. 2011). A formalin model further revealed that 5-HT_4_, 5-HT_6_, and 5-HT_7_ are required for maintaining secondary mechanical allodynia and hyperalgesia (Godinez-chaparro et al. 2012). These findings suggest that 5-HT_2B/2C_ could be involved in inducing mechanical hyperalgesia, whereas 5-HT_4/6/7_ are responsible for maintaining the hyperalgesia. However, the mechanisms of inducing and maintaining mechanical hyperalgesia remain unsolved.

Several lines of evidence have suggested that G_q/11_, G_i_, protein kinase Cε (PKCε), or phospholipase Cβ (PLCβ) is involved in mechanical hyperalgesia (Khasar et al. 1999; Joseph et al. 2007; Dina et al. 2009; Joseph and Levine 2010; Tappe-Theodor et al. 2012). Thus, mechanical hyperalgesia may be mediated by a G_q/11_- or G_i_-PLCβ-PKCε pathway. The in vitro studies found that 5-HT_2_ receptors activate G_q/11_ protein, thus leading to PKC activation (Loric et al. 1995; Lin et al. 2011). so 5-HT_2B/2C_ may mediate 5-HT-induced mechanical hyperalgesia through a G_q/11_-PKC pathway.

Studies in mice lacking transient receptor potential vanilloid 1 (TRPV1) gene revealed that TRPV1 is involved in thermal nociception and hyperalgesia (Caterina et al. 2000; Davis et al. 2000). but several lines of evidence suggest that TRPV1 is involved in mechanical hyperalgesia. TRPV1 antagonists inhibit CFA-, capsaicin, or acid-induced mechanical hyperalgesia (Gavva et al. 2005; Honore et al. 2005; Cui et al. 2006; Chen et al. 2014). Spinal activation of TRPV1 leads to mechanical allodynia, and TRPV1 antagonist can reverse this effect (Kim et al. 2012). Endogenous activation of spinal TRPV1 could be due to G_q/11_-coupled receptors or arachidonic acid metabolites (Gibson et al. 2008; Kim et al. 2009; Kim et al. 2012). 5-HT potentiates TRPV1 function through protein kinase A (PKA) or PKC (Sugiuar et al. 2004; Ohta et al. 2006). Whether TRPV1 is involved in 5-HT-induced mechanical hyperalgesia is unknown.

In this study, we demonstrated that 5-HT-induced mechanical hyperalgesia is regulated by a 5-HT_2B_-G_q/11_-PLCβ-PKCε pathway. Administration of 5-HT strongly enhanced 5-HT- and capsaicin-induced calcium signals in IB_4_-negative neurons, and enhanced signals were regulated by the 5-HT_2B_-G_q/11_-PKCε pathway. Interestingly, 5-HT-induced mechanical hyperalgesia was also inhibited by TRPV1 antagonist or in mice lacking *TRPV1* gene. 5-HT-induced mechanical hyperalgesia may be mediated by a 5-HT_2B_-G_q/11_-PLCβ-PKCε pathway via regulating TRPV1 function.

### Experimental Procedures

#### Animals

Male CD1 mice (8–12 weeks old) were bred and cared for in accordance with the Guide for the Use of Laboratory Animals (National Academy Press, Washington, DC). Animal experimental procedures were approved by the local animal use committee (IACUC, National Central University, Taiwan). TRPV1^−/−^ mice were purchased from the Jackson Laboratory (Bar Harbor, ME, USA) and backcrossed to CD-1 mice for at least ten generations to generate outbred TRPV1^+/−^ mice. The TRPV1^+/+^, TRPV1^+/−^, and TRPV1^−/−^ mice were offspring of a TRPV1^+/−^ intercross. The genotyping primer sequences were 5′-CACGAGACTAGTGAGACGTG/5′-TCCTCATGCACTTCAGGAAA for TRPV1^−/−^ mice and 5′-CCTGCTCAACATGCTCATTG/5′-TCCTCATGCACTTCAGGAAA for TRPV1^+/+^ mice.

#### Agents

5-HT, pertussis toxin (PTX) (Lin et al. 2011; Dina et al. 2009). capsaicin, capsazepine (*N*-[2-(4-chlorophenyl)ethyl]-1,3,4,5-tetrahydro-7,8-dihydroxy-2H-2-benzazepine-2-carbo thioamide) (Zhang et al. 2007). and U73122 (1-[6-[[(17b)-3-methoxyestra-1,3,5(10)-trien-17-yl]amino]hexyl]-1H-pyrrole-2,5-dione) (Hou et al. 2004; Joseph et al. 2007;Lin et al. 2011) were from Sigma (St. Louis, MO). Granisetron hydrochloride (1-methyl-*N*-[(3-endo)-9-methyl-9-azabicyclo[3.3.1]non-3-yl]-1H-indazole-3-carboxamide hydrochloride) (Kayser et al. 2007; Lin et al. 2011). SB206553 hydrochloride (3,5-dihydro-5-methyl-*N*-3-pyridinylbenzo[1,2-b:4,5-b′]dipyrrole-1(2H)-carboxamide hydrochloride)[7], H89 dihydrochloride (*N*-[2-[[3-(4-bromophenyl)-2-propenyl ]amino]ethyl]-5-isoquinolinesulfonamide dihydrochloride) (Zhang et al. 2007; Chen et al. 2008). SQ22536 (9-(tetrahydro-2-furanyl)-9H-purin-6-amine) (Villarreal et al. 2009; Sachs et al. 2009) and RS127445 hydrochloride (4-(4-fluoro-1-naphthalenyl)-6-(1-me thylethyl)-2-pyrimidinamine hydrochloride)(Urtikova et al. 2012) were from Tocris Bioscience (Bristol, UK). PKCεV_1–2_ peptide conjugated with protein transduction domain of TAT protein (CYGRKKRRQRRR-CEAVSLKPT, TAT-PKCεV_1–2_) (Schwarze et al. 1999; Parada et al. 2005; Sachs et al. 2009) was kindly provided by KAI Pharmaceuticals, Inc. (CA, USA). For animal experiments, all drugs or peptides were diluted in saline before injection. For cell experiments, all drugs or peptides were diluted in (4-(2-hydroxyethyl)-1-piperazineethanesulfonic acid) (HEPES)/2-ethanesulfonic acid (MES) buffer (125 mM NaCl, 1 mM KCl, 5 mM CaCl_2_, 1 mM MgCl_2_, 8 mM glucose, 10 mM HEPES, and 15 mM MES, pH 7.6).

#### Behavioral Tests

Pain-behavioral tests were described previously (Lin et al. 2011). Briefly, male CD-1, TRPV1^+/+^, TRPV1^+/−^, and TRPV1^−/−^ mice (8–12 weeks old) were intraplantarly injected with 25 μl 5-HT, receptor antagonists or inhibitors, then underwent animal behavioral tests for withdrawal thresholds to mechanical stimuli applied to the hindpaw (von Frey filaments, Touch-Test; North Coast Medical, Morgan Hill, CA). Mice (*n* = 6 per group) were pre-trained for 2 h each day for 2 days before the test. A series of von Frey fibers (0.6, 1.4, 2.0, 4 g) were applied to the plantar surface of each hindpaw five times at 5-s intervals after injections. The paw withdrawal threshold (PWT) was determined when paw withdrawal was observed in more than three of five applications.

#### Primary Cell Culture

Primary dorsal root ganglia (DRG) were cultured as described (Lin et al. 2011). Briefly, mice were pre-injected with or without 5-HT_2B/2C_ antagonist SB206553, then with 5-HT. After 30 min, mouse lumbar 4–6 DRGs were collected in pre-warmed serum-free Dulbecco’s modified Eagle’s medium (DMEM). After collagenase IA and trypsin treatments, cells were washed in medium and re-suspended in 2 ml serum-free DMEM, then dissociated into single cells by mechanical titration. Cell suspension was slowly dropped into 10 ml serum-free DMEM. After 3∼5 min, the cell suspension on the top (∼10 ml) was collected and centrifuged at 1224×*g* for 5 min. The cell pellet was suspended and mixed in 400 μl DMEM containing 10 % fetal bovine serum (FBS) and seeded on 100 μg/ml poly-l-lysine-coated 24-mm coverslips. After incubation at 37 °C for 2 h, cells were supplemented with 1.5 ml DMEM containing 10 % FBS and maintained at 37 °C for 12 to 14 h before intracellular Ca^2+^ imaging.

#### Intracellular Calcium Imaging

Intracellular calcium imaging was performed as described (Chen et al. 2009; Lin et al. 2011). Primary cultured neurons grown on coverslips were washed once with serum-free medium and pre-incubated at 37 °C with 1.25 μM Fura-2 acetoxymethyl ester (Fura-2-AM; Molecular Probes) for 40 min in HEPES/MES buffer. Coverslips were assembled into culture wells and supplemented with 500 μl HEPES/MES buffer. Cells were stimulated with 500 μl HEPES/MES buffer containing 2-fold concentrations of 5-HT, antagonists, or inhibitors, then underwent intracellular calcium recording with use of a Zeiss inverted microscope equipped with a xenon lamp. Cell images were taken with use of a Zeiss Plan-Apo 63X oil-immersion objective lens. Fura-2-AM fluorescence was measured by 10 Hz alternating-wavelength time scanning, with 340 and 380 nm excitation and 510 nm emission. The fluorescence ratio at two excitation wavelengths (340/380 nm, Ca^2+^-bound Fura-2-AM/free Fura-2-AM) was recorded and analyzed. After recording, cells were stained with IB_4_-FITC conjugates (5 μg/ml) for 15 min and washed with phosphate-buffered saline. IB_4_-FITC-labeled cells were identified by use of a FITC filter at 480 nm excitation and 535 nm emission.

#### In Situ Hybridization and Immunohistochemistry

In situ hybridization and immunohistochemistry were performed as previously described (Lin et al. 2011). Briefly, lumbar 4 DRG tissues were frozen and sectioned in 12-μm-thick slices. Sections were hybridized with 5-HT_2B_-digoxigenin-UTP (dig, Roche)-labeled complementary RNA (cRNA) probes, followed by detection with an alkaline-phosphatase-conjugated anti-dig antibody (Roche). Some sections involved direct staining with IB_4_-FITC conjugates (12.5 μg/ml, Sigma). The specimens were examined by use of a ×20 objective with a fluorescence microscope (Zeiss, Axiovert 200, Germany). The digitized images were captured and the neuron size was measured by MetaMorph software.

#### Statistical Analysis

All data are presented as mean ± SEM. One- or two-way analysis of variance (ANOVA) with post hoc Bonferroni test was used to compare results from multiple groups. The statistically significant levels were set at *p < 0.05, **p < 0.01, and ***p < 0.001.

## Results

### 5-HT-Induced Mechanical Hyperalgesia Is Regulated by the 5-HT_2B_-Gq-PLCβ-PKCε Pathway

**Fig. 1:**
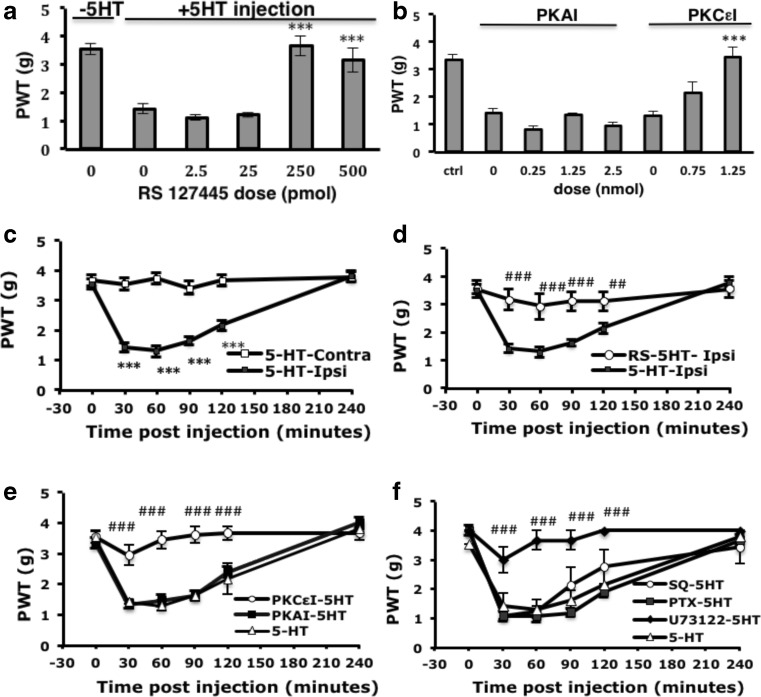
5-HT_2B_ mediates 5-HT-induced mechanical hyperalgesia through phospholipase Cβ (*PLCβ*) and protein kinase Cε (*PKCε*). **a** Wild-type CD1 mice (8–12 weeks old) were intraplantarly injected with different doses of 5-HT_2B_ antagonist (RS127445 (*RS*)) before 5-HT injection. Mechanical tests were performed and the threshold of paw withdrawal (*PWT*) was measured at 30 min. Data are mean ± SEM PWT of total tested mice (*n* ≥ 6 per group). Comparisons between inhibitor-injected groups and inhibitor-uninjected groups were done by one-way ANOVA with a post hoc Bonferroni test. ***p < 0.001. **b** Mice were intraplantarly injected with 25 μl of different doses of PKA inhibitor (*PKAI*; H89) or PKCε inhibitor (*PKCεI*; TAT-PKCεV_1–2_) before 5-HT injection. The PWT was measured at 30 min (for PKAI) or 60 min (for PKCεI) after 5-HT injection. Inhibitor-injected groups were compared with inhibitor-uninjected groups by one-way ANOVA with a post hoc Bonferroni test. ***p < 0.001. **c** Mice were injected with 5-HT, followed by mechanical tests at different times. Ipsilateral PWTs were compared with contralateral PWTs by two-way ANOVA with a post hoc Bonferroni test. ***p < 0.001. **d** Mice were pre-injected with RS (20 μM) before 5-HT injection, followed by mechanical tests at different times. Comparisons between the ipsilateral PWTs of 5-HT-injected and RS/5-HT-injected animals were done by two-way ANOVA with a post hoc Bonferroni test. ^*##*^ *p* < 0.01; ^###^ *p* < 0.001. **e** Mice were pre-injected with PKCεI (50 μM) or PKAI (50 μM) before 5-HT injection. PWT for ipsilateral paw was measured at different times. The ipsilateral PWTs of 5-HT/inhibitor- were compared with the ipsilateral PWTs of 5-HT-injected animals by two-way ANOVA with a post hoc Bonferroni test. ^##^ *p* < 0.01; ***p < 0.001; ^###^ *p* < 0.001. **f** Mice were pre-injected with AC inhibitor (SQ22536 (*SQ*), 1 mM), G_i_ inhibitor (pertussis toxin (*PTX*), 4 ng/μl) or PLCβ inhibitor (U73122, 500 μM) before 5-HT injection. PWT for ipsilateral paw was measured at different times. Comparisons between ipsilateral PWTs of 5-HT/inhibitor- and ipsilateral PWTs of 5-HT-injected animals (*number sign*), were done by two-way ANOVA with a post hoc Bonferroni test. ^###^ *p* < 0.001

We previously found that 5-HT-induced mechanical hyperalgesia was inhibited by the 5-HT_2B_/_2C_ antagonist SB206553 (Lin et al. 2011). To determine whether 5-HT_2B_ or 5-HT_2C_ mediated the 5-HT-induced mechanical hyperalgesia, we administered the selective antagonist of 5-HT_2B_ (RS127445). The effect doses for RS127445 were 0.25 and 0.5 nmol (Fig. 1a). In agreement with the previous study (Lin et al. 2011). 5-HT injection into the mouse hindpaw induced mechanical hyperalgesia within 30 min, which disappeared at 4 h (Fig. 1c). Administration of RS127445 at the dose of 0.5 nmol completely inhibited mechanical hyperalgesia induced by 5-HT (Fig. 1d). It is confirmed that 5-HT_2B_ mediated 5-HT-induced mechanical hyperalgesia. 5-HT binding to 5-HT_2B_ receptors activated G_q_ protein, thus activating PLC_β_, then PKC and increased intracellular calcium concentration (Loric et al. 1995; Lin et al. 2011). To further confirm that the G_q_-PLC_β_-PKC pathway is involved in 5-HT_2B_-mediated mechanical hyperalgesia, we tested the doses of PKCε or PKA inhibitors. PKCε or PKA inhibitors were injected before 5-HT injection, and mechanical hyperalgesia was tested at 30 min after 5-HT injection. PKA inhibitor (H89) did not block 5-HT-induced hyperalgesia even in 2.5 nmol dose (Fig. 1b), but 1.25 nmol of PKA inhibitor was able to reduce CFA-induced mechanical hyperalgesia (data not shown). The dose of 1.25 nmol for PKA was used in the following experiments. PKCε inhibitor (PKCεI, peptide V_1–2_) reduced 5-HT-induced hyperalgesia at the dose of 0.75 nmol and completely block hyperalgesia at 1.25 nmol. The dose of 1.25 nmol for PKCεI was used in the following experiments (Fig. 1b). We then examined the effects of PKAI and PKCεI at different times after 5-HT injection. With injection of 1.25 nmol PKCεI before 5-HT injection, the hyperalgesia to mechanical stimuli was completely inhibited within 30 min (3.67 ± 0.33 g on the PKCεI-injected ipsilateral paw and 1.42 ± 0.15 g on the 5-HT-injected ipsilateral paw, Fig. 1e) and the inhibition lasted at least 4 h. Similar experiments blocking PKA with H89 could not inhibit 5-HT-induced mechanical hyperalgesia (1.33 ± 0.07 g on the PKAI-injected ipsilateral paw Fig. 1e). We then examined the involvement of G-protein signaling in 5-HT-induced mechanical hyperalgesia. The effective doses for SQ22536, U73122, and PTX were first tested in CFA model (data not shown). The dose of 5.1 μg of adenylyl cyclase (AC) inhibitor SQ22536, 5.8 μg of PLCβ inhibitor (U73122), or 100 ng of Gi/o protein inhibitor (PTX) was injected into mouse paw. Only PLCβ blocker but not AC or Gi/o protein inhibitor could inhibit 5-HT-induced mechanical hyperalgesia (Fig. 1f). Therefore, 5-HT-induced mechanical hyperalgesia was mediated by the 5-HT_2B_-G_q_-PLCβ-PKCε pathway.

### Enhanced 5-HT-Induced Calcium Signals Are Regulated by 5-HT_2B_-PLCβ-PKCε Signaling

**Fig. 2:**
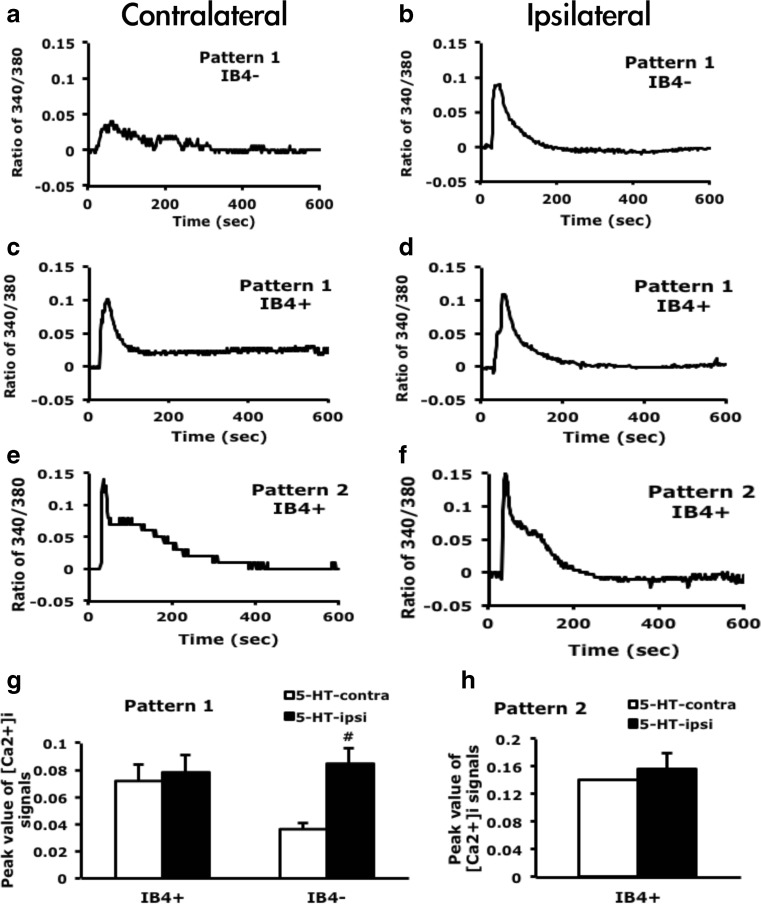
IB_4_-negative neurons show enhanced calcium signals after 5-HT injection. Lumbar 4–6 dorsal root ganglia (*DRG*) ipsilateral or contralateral to the 5-HT-injected mouse paw were cultured for 12 h, then stimulated with 5-HT (1 μM), and intracellular calcium change was recorded for 600 s. After recording, neurons were pulse labeled with IB_4_-FITC (5 μg/ml) for 10 min. Time-dependent mean calcium increase in IB_4_-negative (*IB*_*4*_ *−*) neurons (**a**, **b**) and IB_4_-positive (*IB*_*4*_ *+*) neurons (**c**–**f**). The peak values were represented in the histograms (**g**, **h**). Comparisons between ipsilateral and contralateral DRG neurons of 5-HT-injected animals were done by two-way ANOVA with a post hoc Bonferroni test. ^#^ *p* < 0.05

**Fig. 3:**
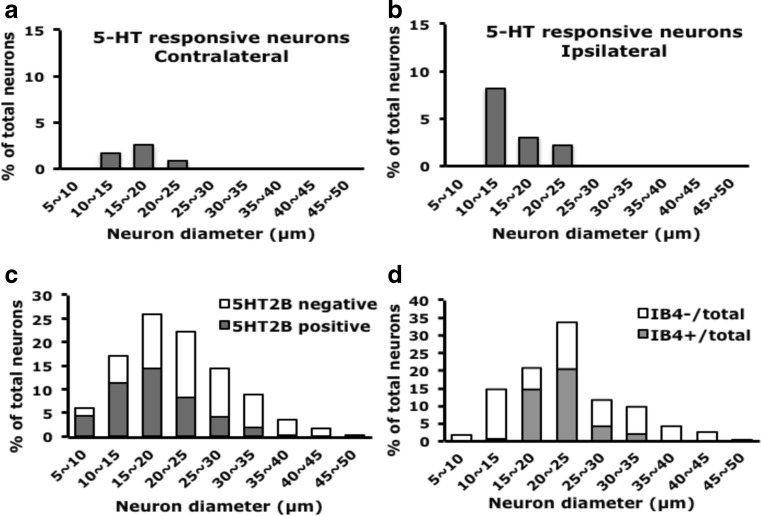
5-HT-responsive neurons are small-diameter neurons. **a**, **b** Lumbar 4–6 DRG ipsilateral or contralateral to the 5-HT-injected mouse paw were cultured for 12 h, then stimulated with 5-HT (1 μM), and intracellular calcium change was recorded for 600 s. The diameter of neurons was measured by MetaMorph software. Histograms show proportion of 5-HT-responsive neurons to total recorded neurons. **c**, **d** Lumbar DRG tissues were sectioned and hybridized with DIG-labeled 5-HT_2B_ antisense cRNA probes (**c**) or directly stained with IB_4_-FITC conjugates (**d**). The diameter of neurons was measured by MetaMorph software. Histograms show proportion of **c** 5-HT_2B_-positive neurons to total neurons and **d** IB_4_-positive or IB_4_-negative subpopulations to respective total neurons

In a previous study (Lin et al. 2011). we found that 5-HT-induced calcium signals in cultured DRG neurons had two patterns: (1) transient increase and (2) transient increase and sustained return. Only pattern 1 was significantly enhanced after 5-HT injection. However, the subsets of DRG neurons with the enhanced signals remained unknown. We cultured DRG neurons from mice after 5-HT injection and examined 5-HT-induced calcium signals. Consistent with previous findings, 5-HT-induced calcium signals had 2 patterns. Pattern 2 occurred only in IB_4_-positive neurons, but pattern 1 appeared in both IB_4_-positive and IB_4_-negative neurons (Fig. 2a–f). The peak of pattern 2 signals did not change after 5-HT injection (Fig. 2h). After 5-HT injection, pattern 1 signals were 3-fold enhanced in IB_4_-negative neurons (0.035 ± 0.021 contralateral side vs. 0.106 ± 0.022 ipsilateral side), but remained unchanged in IB_4_-positive neurons (Fig. 2g). Although pattern 1 signals in IB_4_-positive neurons were not enhanced, the number of 5-HT responsive neurons was increased (2.5 % contralateral side vs. 5.2 % ipsilateral side). The number of 5-HT-responsive IB_4_-negative neurons was also increased (1.7 vs. 5.9 %). 5-HT-responsive neurons were 10 to 25 μm in diameter, with most being 10 to 15 μm in diameter and increased in proportion after 5-HT injection (total responsive neurons are 5.1 % on contralateral and 13.3 % on ipsilateral sides, Fig. 3a, b). In situ hybridization revealed that neurons expressing 5-HT_2B_ were 5 to 35 μm in diameter, with most 10 to 25 μm (Fig. 3c). The size of 5-HT_2B_-expressing neurons was correlated with the size of 5-HT-responsive neurons, which suggested that 5-HT_2B_ mediated 5-HT-induced signals in small-diameter neurons (10 to 25 μm). Immunostaining of DRG sections revealed that among the 10- to 25-μm diameter neurons, IB_4_-positive immunoreaction was mainly located in neurons 15 to 25 μm in diameter (Fig. 3d). This finding was consistent with IB_4_ staining of cultured DRG neurons. 5-HT-responsive neurons <15 μm were mainly IB_4_ negative. 5-HT-enhanced calcium signals were also in IB4-negative population (Fig. 2h).

**Fig. 4:**
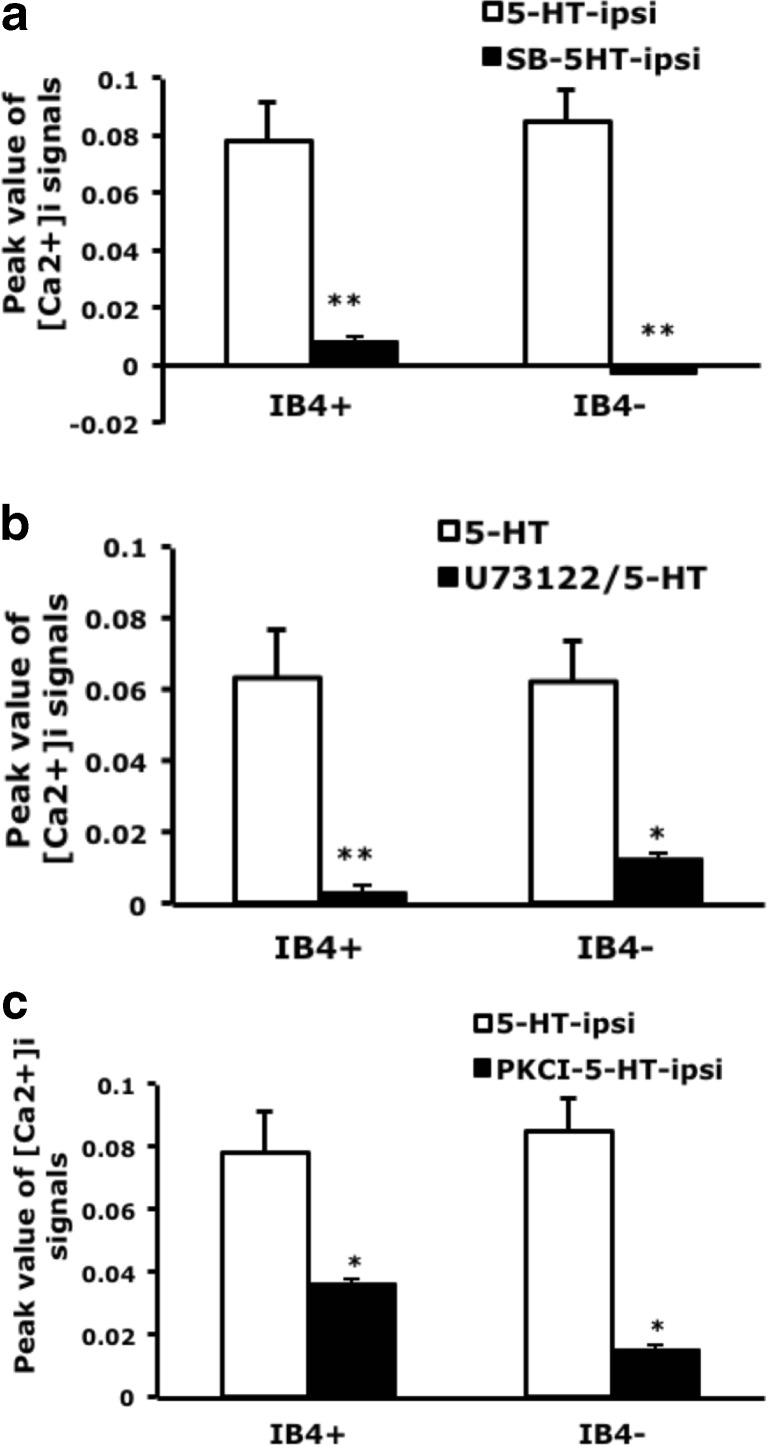
Pattern 1 signals are inhibited by 5-HT_2B/2C_ PLCβ and PKCε inhibitors. **a** Mice were injected with 5-HT or SB206553 (10 μM)/5-HT. At 30 min, lumbar 4–6 DRG ipsilateral to the 5-HT-injected paw and SB206553/5-HT-injected paw were taken and cultured for 12 h. Neurons were then stimulated with 5-HT (1 μM), and intracellular calcium change was recorded for 600 s. After recording, the neurons were pulse-labeled with IB_4_-FITC (5 μg/ml) for 10 min. Data are mean ± SEM peak values of [Ca^2+^]_i_ signals (∼35 s after the addition of 5-HT). Comparison between 5-HT-injected and SB206553-5-HT-injected neurons was done by two-way ANOVA with a post hoc Bonferroni test. **p < 0.01. **b** DRG neurons were pre-incubated with the PLCβ inhibitor U73122 (5 μM) for 5 min, then stimulated with 5-HT (1 μM); calcium signals were recorded for 400 s. U73122-treated groups were compared with untreated groups by two-way ANOVA with a post hoc Bonferroni test. *p < 0.05; **p < 0.01. **c** Mice were injected with 5-HT or PKCεI (50 μM)/5-HT. Lumbar 4–6 DRG were cultured and stimulated with 5-HT (1 μM); calcium signals were recorded for 200 seconds. Comparison between 5-HT-injected and PKCεI-5-HT-injected groups was done by two-way ANOVA with a post hoc Bonferroni test. *p < 0.05

Consistent with previous findings (Lin et al. 2011). injection of a 5-HT_2B/2C_ antagonist inhibited pattern 1 signals and the transient signals in pattern 2; pattern 1 signals were completely inhibited in both IB_4_-positive and IB_4_-negative neurons (Fig. 4a). The number of 5-HT-responsive neurons was also decreased (1.3 % with SB206553 and 5-HT injection vs. 13 % with 5-HT injection).

To examine whether 5-HT-induced pattern 1 calcium signals depend on PLCβ, we treated neurons with the PLCβ inhibitor, U73122. Signal pattern 1 was inhibited by the addition of U73122 in both IB_4_-positive and IB_4_-negative neurons (Fig. 4b), suggesting that pattern 1 calcium signals are regulated by PLCβ. We then used the PKCε blocker PKCεI to examine 5-HT-induced calcium signals. PKCεI completely inhibited 5-HT-induced calcium signals in IB_4_-negative neurons, but only partially blocked calcium signals in IB_4_-positive neurons (Fig. 4c).

### Enhanced 5-HT-Induced Calcium Signals Are Regulated by a 5-HT_2B_-PKCε-Dependent Pathway

**Fig. 5:**
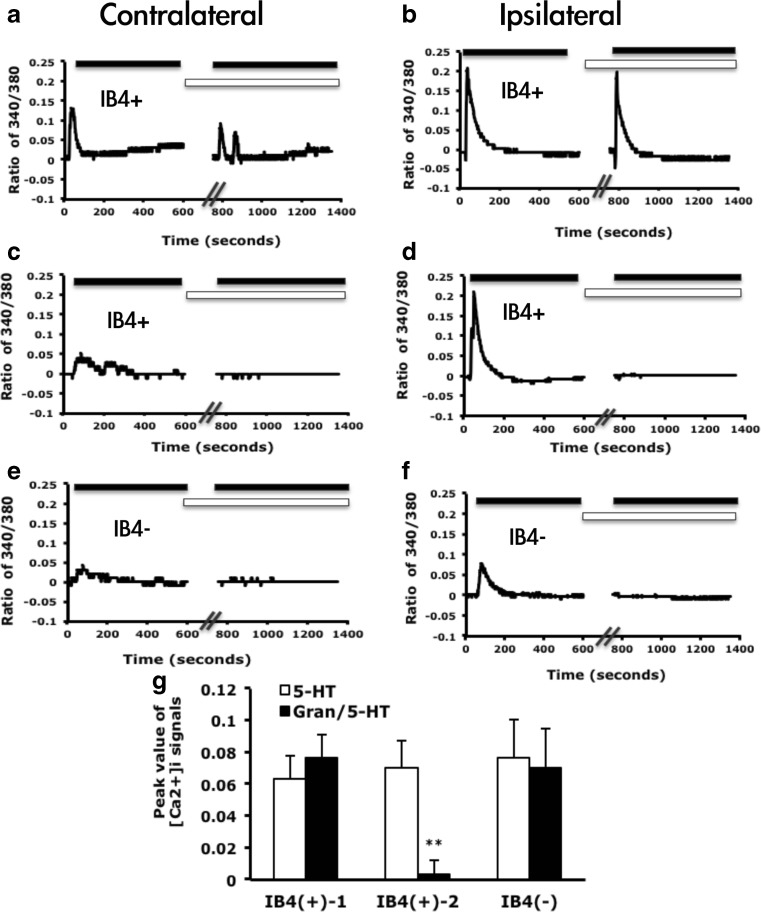
5-HT-induced calcium signals have different sensitivities to EGTA. Lumbar 4–6 DRG ipsilateral or contralateral to the 5-HT-injected mouse paw were cultured for 12 h, then stimulated with 5-HT (1 μM), and intracellular calcium change was recorded for 600 s. After a washing, neurons were pre-incubated with EGTA (2 mM) for 10 min (**a**–**f**) or with 5-HT_3_ antagonist (Granisetron (*Gran*), 1 μM, **g**) for 5 min, then stimulated with 5-HT (1 μM); calcium signals were recorded for 1400 or 400 s, respectively. Neurons were then pulse labeled with IB_4_-FITC (5 μg/ml) for 10 min. Time-dependent mean calcium increase in IB_4_-positive (**a**–**d**) and IB_4_-negative neurons (**e**–**f**). *White bars* indicate the presence of EGTA, *black bars* indicate the time for addition of 5-HT (1 μM). **g** Granisetron-treated responses were divided into two types: unchanged (type 1) and decreased (type 2). Comparison between 5-HT treatment alone and Granisetron/5-HT-treated groups was done by one-way ANOVA with a post hoc Bonferroni test. *p < 0.05; **p < 0.01; ***p < 0.001

In IB_4_-positive neurons, 5-HT-induced calcium signaling remained unchanged in some 5-HT-responsive neurons with EGTA treatment to remove extracellular calcium (Fig. 5a, b), but was completely inhibited in some other neurons (Fig. 5c, d). In IB_4_-negative neurons, 5-HT-induced signals were sensitive to removal of extracellular calcium (Fig. 5e, f). Therefore, calcium signals from some 5-HT-responsive IB_4_-positive neurons are from the endoplasmic reticulum (ER) and are directly induced by 5-HT_2_ activation. However, calcium signals from other 5-HT-responsive IB_4_-positive neurons and all 5-HT-responsive IB_4_-negative neurons are from extracellular pools through unknown channels. It may explain that 5-HT-induced calcium signals were only partially inhibited by PKCεI in IB_4_-positive neurons because 5-HT_2B_-mediated calcium release from ER is insensitive to EGTA. In IB_4_-negative neurons, 5-HT-induced calcium signals were from extracellular pools and regulated by PKCε.

One possibility for calcium influx is due to 5-HT_3_. In IB_4_-negative neurons, the addition of a 5-HT_3_ antagonist granisetron could not inhibit 5-HT-induced calcium signals (Fig. 5g). In IB_4_-positive neurons, 5-HT-induced calcium signals were inhibited by the 5-HT_3_ antagonist in some neurons but remained unchanged in some neurons (Fig. 5g). The partial inhibition of granisetron in IB_4_-positive neurons suggested that calcium signals were partially sensitive to EGTA.

Thus, in IB_4_-positive neurons, some 5-HT-induced calcium signals are released from an internal Ca^2+^ store that is directly induced by 5-HT_2B/2C_ activation and some from extracellular influx through 5-HT_3_ or other calcium channels regulated by a 5-HT_2B/2C_-PKCε pathway. In IB_4_-negative neurons, all 5-HT-induced calcium signals are from Ca^2+^ influx and are regulated by a 5-HT_2B/2C_-PKCε pathway.

### TRPV1 Is Involved in 5-HT-Induced Mechanical Hyperalgesia

**Fig. 6:**
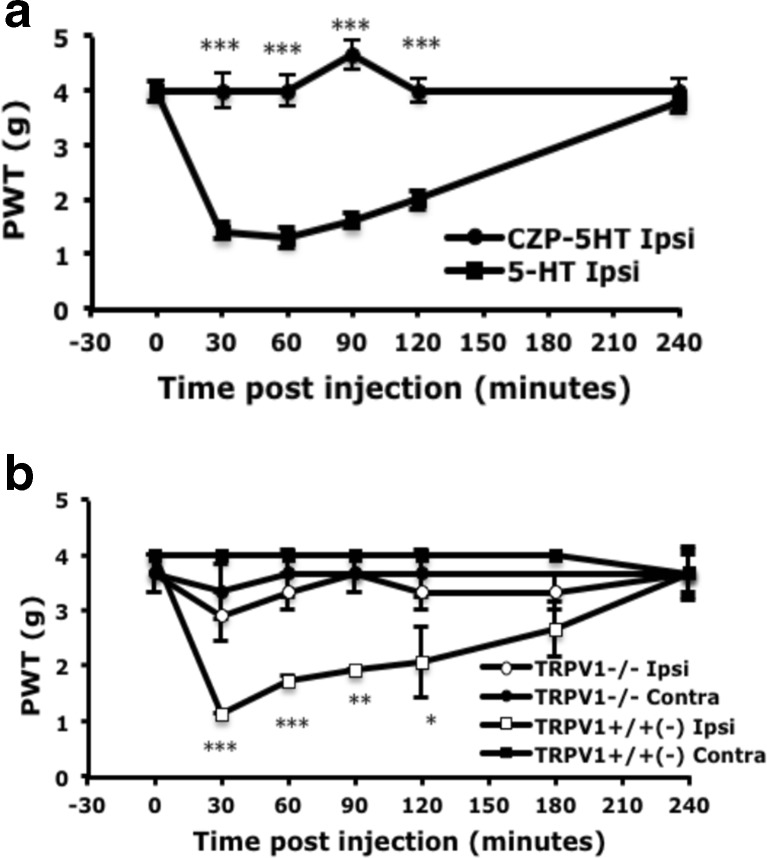
5-HT-induced mechanical hyperalgesia is inhibited by peripheral injection of TRPV1 antagonist or in mice lacking TRPV1. **a** Wild-type CD1 mice (8–12 weeks old) were pre-injected with or without 25 μl TRPV1 antagonist (capsazepine (*CZP*), 10 μM), then with 25 μl 5-HT (10 μM). **b** TRPV1^+/+^, TRPV1^+/−^, and TRPV1^−/−^ mice (8–16 weeks old) were injected with 25 μl 5-HT (10 μM). The threshold of paw withdrawal (*PWT*) was measured before (*t* = 0) and after injection. Data are mean ± SEM PWT of total tested mice (*n* = 6 per group). Data for TRPV1^+/+^ and TRPV1^+/−^ mice are grouped together. Comparison between 5-HT-injected and CZP-5HT-injected animals or between ipsilateral side of TRPV1^+/+^ and TRPV1^−/−^ animals was done by two-way ANOVA with a post hoc Bonferroni test. *p < 0.05; **p < 0.01; ***p < 0.001

Previous studies suggested that TRPV1 function is enhanced by 5-HT (Sugiuar et al. 2004; Ohta et al. 2006) and TRPV1 is involved in capsaicin-, acid-, or CFA-induced mechanical hyperalgesia (Gavva et al. 2005; Honore et al. 2005; Cui et al. 2006; Chen et al. 2014). Thus, we examined whether TRPV1 is involved in 5-HT-induced mechanical hyperalgesia. The administration of the TRPV1 antagonist capsazepine (0.25 nmol) before 5-HT injection completely inhibited hyperalgesia to mechanical stimuli within 30 min and lasted at least 4 h (Fig. 6a). We further validated the role of TRPV1 in 5-HT-induced mechanical hyperalgesia using TRPV1-deficient mice. 5-HT injection induced mechanical hyperalgesia in TRPV1^+/−^ and TRPV1^+/+^ but not TRPV1^−/−^ mice (Fig. 6b). The results suggested that TRPV1 participates 5-HT-induced mechanical hyperalgesia.

**Fig. 7:**
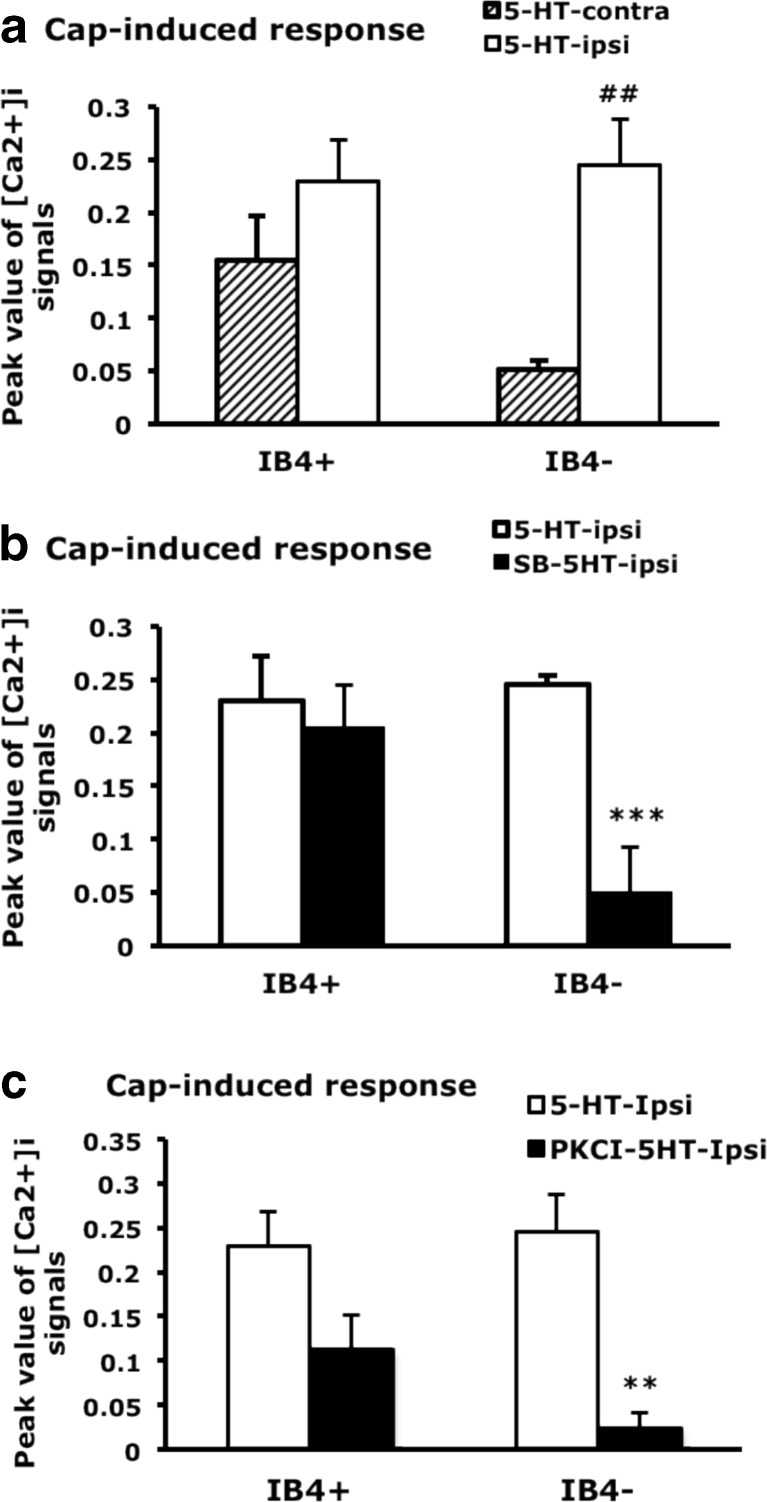
Capsaicin-induced calcium influx is enhanced by 5-HT and inhibited by 5-HT_2B/2C_ antagonist and PKCε blocker. Lumbar 4–6 DRG ipsilateral or contralateral to the 5-HT-injected, SB206553-5-HT, or PKCεI-5-HT-injected mouse paw were cultured for 12 h, then stimulated with capsaicin (100 nM), and intracellular calcium change was recorded for 600 s. After recording, neurons were pulse labeled with IB_4_-FITC (5 μg/ml) for 10 min. Peak values of [Ca^2+^]_i_ signals (approximately 35 s after the addition of agonists) represent data points as indicated. Comparisons between ipsilateral and contralateral sides of 5-HT-injected animals (*number sign*), between ispilateral side of 5-HT-injected and SB206553-5-HT-injected animals (*asterisk*) or between ispilateral side of 5-HT-injected and PKCεI-5-HT-injected animals (*asterisk*) were done by two-way ANOVA with a post hoc Bonferroni test. ^##^ *p* < 0.01; **p < 0.01; ***p < 0.001

We next examined whether TRPV1 function is regulated by 5-HT_2B_-mediated signaling. Capsaicin-induced calcium signals were greater in IB_4_-positive than -negative neurons (0.155 ± 0.042 vs. 0.051 ± 0.09 contralateral side; Fig. 7a), which agrees with previous findings (Petruska et al. 2000, 2002; Liu et al. 2004). After 5-HT injection, capsaicin-induced calcium signals were increased in IB_4_-negative neurons (0.245 ± 0.044 ipsilateral side vs. 0.051 ± 0.09 contralateral side) but not IB_4_-positive neurons (Fig. 7a). Injection of 5-HT_2B/2C_ antagonist inhibited the 5-HT-enhanced capsaicin-evoked signals in IB_4_-negative but not IB_4_-positive neurons (Fig. 7b), which suggests that 5-HT-enhanced TRPV1 function in IB_4_ negative is regulated by 5-HT_2B/2C_. We further examined whether PKCε regulates enhanced TRPV1 function. Capsaicin-evoked signals were also inhibited by PKCεI in IB_4_-negative neurons (Fig. 7c). Accordingly, these results suggests that 5-HT enhances TRPV1 function through the 5-HT_2B_-PKCε pathway.

## Discussion

**Fig. 8:**
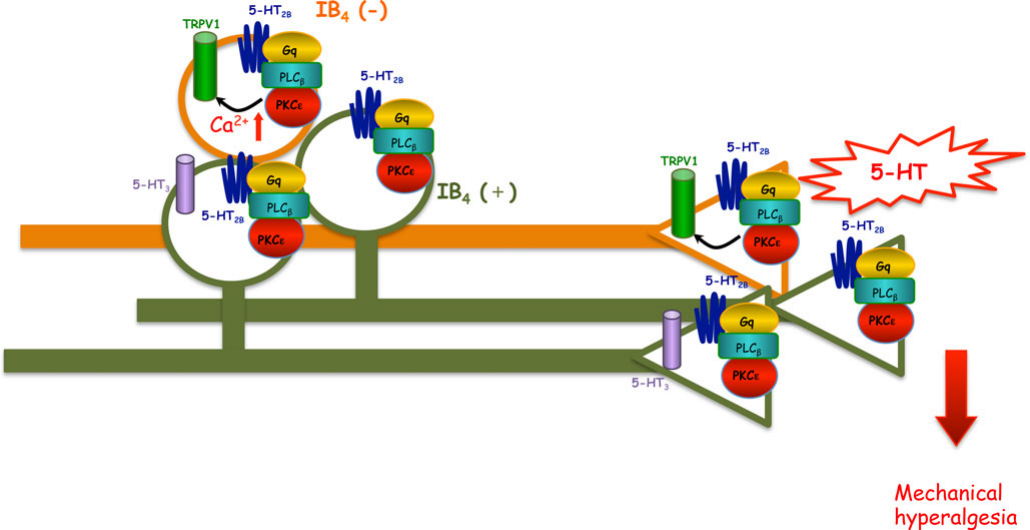
Putative model of 5-HT-induced mechanical hyperalgesia through 5-HT_2B_-mediated pathways. In the periphery of IB_4_-negative neurons, 5-HT_2B_ responds to 5-HT to activate G_q_ protein and PLCβ, thus activating PKCε. PKCε activation enhances TRPV1 function, thereby leading to mechanical hyperalgesia. In the cell body of IB_4_-negative neurons, peripheral 5-HT stimulation increases the number of 5-HT-responsive neurons and 5-HT-evoked calcium signals. Enhanced calcium signals could be due to regulation of TRPV1 by 5-HT_2B_-PLCβ-PKCε pathway. In the periphery of IB_4_-positive neurons, 5-HT_2B_ responds to 5-HT to activate G_q_ protein, PLCβ, and PKCε, thereby leading to mechanical hyperalgesia. In the cell body of IB_4_-positive neurons, peripheral 5-HT stimulation increases the number of 5-HT-responsive neurons

Our previous findings suggested that 5-HT_2B/2C_ antagonist inhibited 5-HT-induced mechanical hyperalgesia. In this study, we demonstrated that 5-HT_2B_-PLCβ-PKCε pathway and TRPV1 function are involved in 5-HT-induced mechanical hyperalgesia because administration of antagonists for 5-HT_2B_, PLCβ, PKCε, or TRPV1 inhibited 5-HT-induced mechanical hyperalgesia. In ipsilateral DRG neurons, 5-HT injection increased 5-HT or capsaicin-induced calcium signals mainly in IB_4_-negative neurons, which was regulated by the 5-HT_2B_-PLCβ-PKCε pathway. 5-HT injection caused unilateral hyperalgesia in injected site and facilitated 5-HT signaling in ipsilateral DRG. It is likely that plastic changes occur only in injected peripheral terminal of nociceptors and in ipsilateral DRG, resulting in peripheral sensitization, inducing ipsilateral hyperalgesia. Because no facilitated 5-HT responsiveness occurred in contralateral DRG, it is indicated that no signal was passed to the contralateral side. Thus only unilateral hyperalgesia was observed after 5-HT injection. A possible mechanism is that initial insult (5-HT) induces activation of 5-HT_2B_-PLCβ-PKCε in the peripheral terminal of the nociceptor. Activation of this pathway could relieve TRPV1 from PIP_2_ inhibition (Chuang et al. 2001) to produce peripheral sensitization that increases the number of neurons responding to 5-HT and facilitates 5-HT or capsaicin-induced signaling in IB_4_-negative neurons (Fig. 8). Thus, 5-HT-induced mechanical hyperalgesia may be mainly mediated by 5-HT_2B_-G_q_-PLCβ-PKCε signaling by regulating TRPV1 function.

We previously suggested that 5-HT_2B_ mediates 5-HT-induced mechanical hyperalgesia using 5-HT_2B/2C_ antagonist, SB206553 (Lin et al. 2011). Interestingly, Urtikova et al. (2012) found antinociceptive effects of 5-HT_2B_ on neuropathic pain in rats, using 5-HT_2B_ selective antagonist RS127445. We used RS127445 in our mouse model but found that 0.25 and 0.5 nmol RS1277445 blocked 5-HT-induced mechanical hyperalgesia. These results confirmed our previous suggestion that 5-HT_2B_ plays a pro-nociceptive role to mediate 5-HT-induced mechanical hyperalgesia, although 5-HT_2C_ involvement cannot be completely ignored given that a number of studies have shown that 5-HT_2C_ receptor has pro-nociceptive or antinociceptive effects (Jeong et al. 2004; Nakajima et al. 2009; Nakai et al. 2010). Previous findings suggested that PLCβ, G_q/11_, G_i_, PKCε are involved in carrageenan- or PGE_2_-inudced mechanical hyperalgesia (Khasar et al. 1999; Joseph et al. 2007; Dina et al. 2009; Tappe-Theodor et al. 2012). Blocking PLCβ or PKCε but not G_i_ or AC specifically inhibited 5-HT-induced mechanical hyperalgesia, which further indicates that the G_q_-PLCβ-PKCε pathway is involved in 5-HT-induced mechanical hyperalgesia. Activation of 5-HT_2B_ induces Gq-PLCβ-PKCε signaling (Loric et al. 1995; Lin et al. 2011). The dependency of 5-HT-induced mechanical hyperalgesia on the G_q_-PLCβ-PKCε pathway gives further evidence of the involvement of 5-HT_2B_. Clearly, 5-HT_2B_ has pro-nociceptive rather than antinociceptive effects in 5-HT-induced mechanical hyperalgesia. The antinociceptive roles of 5-HT_2B_ on neuropathic pain may be influenced by other inflammatory mediators or immune cells a suggested previously (Urtikova et al. 2012).

Administration of 5-HT into mice increased the number of DRG neurons responding to 5-HT (Fig. 3) in both IB_4_-positive and IB_4_-negative neurons, suggesting that both types of neurons are involved in 5-HT-induced pain. However, 5-HT-evoked transient calcium signals (pattern 1) were enhanced in only IB_4_-negative neurons. 5-HT-evoked calcium signals were greater in IB_4_-positive than -negative neurons before 5-HT injection, which may explain the enhanced calcium signals found only in IB_4_-negative neurons. IB_4_-positive and IB_4_-negative neurons may play distinct roles in 5-HT-induced pain. Injection of 5-HT_2B/2C_ antagonist inhibited pattern 1 transient signals in both types of neurons. Given that the 5-HT_2B/2C_ antagonist blocks only 5-HT-induced mechanical hyperalgesia (Lin et al.,^2011^). pattern 1 calcium signals inhibited by 5-HT_2B/2C_ antagonist may be involved in 5-HT-induced mechanical hyperalgesia. Accordingly, both IB_4_-positive and IB_4_-negative neurons are involved, although pattern 1 signals in IB_4_-positive neurons were not enhanced. Stucky and Lewin (1999) suggested that IB_4_-negative neurons have lower action potential (AP) threshold and shorter AP duration than IB_4_-positive neurons: IB_4_-negative neurons could be essential in transducing information about stimuli, but IB_4_-positive neurons could be more important for transmission of information at the first central synapse. 5-HT-induced mechanical hyperalgesia is not a chronic effect (lasts 2 h). Therefore, 5-HT injection greatly enhanced calcium signals in IB_4_-negative neurons, which may be responsive for transducing 5-HT stimuli, rather than in IB_4_-positive neurons, which may be important in synaptic transmission. Both types of neurons participate in mechanical hyperalgesia, so the signals can be inhibited by 5-HT_2B/2C_ antagonist.

In IB_4_-negative neurons, inhibition of 5-HT_2B_, PKCε and PLCβ completely blocked 5-HT-induced pattern 1 calcium signals, so pattern 1 signals may be regulated by the 5-HT_2B_-PLCβ-PKCε pathway. Pattern 1 calcium increase was also sensitive to the addition of EGTA, which indicates that the increase was due to calcium influx through calcium channels. However, the calcium influx was not through 5-HT_3_ receptor because 5-HT_3_ receptor antagonist, granisetron, did not block the calcium influx. 5-HT-induced calcium influx may occur through other calcium channels in IB_4_-negative neurons. In contrast, IB_4_-positive neurons have more complicated responses. 5-HT-evoked calcium signals were completely inhibited by 5-HT_2B/2C_ antagonist and PLCβ, but partially inhibited by PKCεI, suggesting that calcium signals were partially regulated by 5-HT_2B_-PLCβ-PKCε pathways. Since calcium signals in some IB_4_-positive neurons were sensitive to EGTA, it indicated that the part of calcium signals was from calcium influx. Calcium signals unregulated by PKCε could directly result from 5-HT_2B/2C_ activation that leads to calcium release from ER store, which are insensitive to EGTA. Calcium signals regulated by PKCε could be from voltage-gated calcium channels or other calcium channels. Interestingly, 5-HT_3_ antagonist specifically inhibited calcium signals of some IB_4_-positive neurons. Thus, calcium influx regulated by 5-HT_2B_-PLCβ-PKCε pathway is more likely from 5-HT_3_ channel. 5-HT_3_ was previously found not involved in 5-HT-induced mechanical hyperalgesia (Lin et al. 2011) but is involved in 5-HT-induced thermal hyperalgesia (Loyd et al. 2012). Although 5-HT_3_ antagonist cannot completely block 5-HT-induced mechanical hyperalgesia, it shortens the hyperalgesia response (Lin et al. 2011). which agrees with our finding that pattern 1 signals in some IB_4_-positive neurons were mediated by 5-HT_3_ because such neurons are important in synaptic transmission (Stucky and Lewin 1999). 5-HT_3_ could be involved in maintaining mechanical hyperalgesia, which is also regulated by 5-HT_2B_-PLCβ-PKCε pathway.

Serotonin has been shown to enhance TRPV1 functions (Sugiuar et al. 2004; Ohta et al. 2006). As expected, we found that 5-HT injection enhanced capsaicin-evoked calcium signals but only in IB_4_-negative not IB_4_-positive neurons. Interestingly, capsaicin-evoked calcium signals were greater in IB_4_-positive than IB_4_-negative neurons before 5-HT injection, which is consistent with the study in rats (Liu et al. 2004) but not mice (Dirajlal et al. 2003; Breese et al. 2005). The difference could be due to the outbred mice we used. The increased capsaicin-evoked calcium signals in IB_4_-negative neurons explain the effect of 5-HT injection only in IB_4_-negative neurons. The capsaicin-evoked signals in IB_4_-negative neurons were greatly inhibited by 5-HT_2B/2C_ antagonism and PKCε blockage, which suggests that 5-HT-enhanced capsaicin-evoked calcium signals were regulated by the 5-HT_2B/2C_-PKCε pathway. Capsaicin-sensitive IB_4_-negative neurons have lower mechanical threshold and larger mechanical evoked currents than capsaicin-sensitive IB_4_-positive neurons (Drew et al. 2002). Therefore, 5-HT-enhanced capsaicin-evoked calcium signals in IB_4_-negative neurons may be important in 5-HT-induced mechanical hyperalgesia. Administration of a TRPV1 antagonist before 5-HT injection in mice specifically inhibited 5-HT-induced mechanical hyperalgesia. Mice lacking the TRPV1 gene did not show mechanical hyperalgesia in response to 5-HT stimuli, which suggests that TPRV1 is involved in 5-HT-induced mechanical hyperalgesia. TRPV1 involvement in mechanical hyperalgesia is surprising but not impossible. Several lines of evidence have proposed that TRPV1 antagonists inhibit capsaicin-, acid-, or CFA-induced mechanical hyperalgesia (Gavva et al. 2005; Honore et al. 2005; Cui et al. 2006; Chen et al. 2014). Kim et al. (2012) proposed that spinal TRPV1 plays critical roles in mediating neuropathic mechanical allodynia. Activation of spinal TRPV1 could be due to G_q/11_-coupled receptors or arachidonic acid (AA) metabolites (Gibson et al. 2008; Kim et al. 2009; Kim et al. 2012). Given that 5-HT_2_ receptor activation can activate phospholipase A2, thus leading to AA release (Tournois et al. 1998). peripheral 5-HT_2B_ activation by 5-HT may induce AA release to activate peripheral TRPV1, thus affecting mechanical hyperalgesia.

Although calcium signals regulated by 5-HT_2B_ are critical for 5-HT-induced mechanical hyperalgesia, sodium currents may also have important roles in mechanical hyperalgesia. Several lines of evidence have suggested that 5-HT increases tetrodotoxin-resistant (TTX-R) I_Na_ currents (Gold et al. 1996). Na_v_1.8 (a TTX-R channels) is related to inflammatory hyperalgesia (Abrahamsen et al. 2008; Yen et al. 2009). and PKC can modulate TTX-R I_Na_ currents (Gold et al. 1998; Cang et al. 2009). Therefore, 5-HT_2B_-G_q_-PLCβ-PKCε signaling could regulate voltage-gated Na^+^ channels to affect mechanical hyperalgesia.

In conclusion, our study demonstrates that 5-HT_2B_-G_q_-PLCβ-PKCε signaling and TRPV1 function are involved in 5-HT-induced mechanical hyperalgesia. Peripheral 5-HT stimulation increased the responsiveness of DRG neurons to 5-HT. The number of both IB_4_-positive and IB_4_-negative neurons responding to 5-HT were increased. 5-HT or capsaicin-evoked calcium signals were increased in IB_4_-negative neurons by 5-HT_2B_-G_q_-PLCβ-PKCε pathway. Accordingly, 5-HT-induced mechanical hyperalgesia may be mainly mediated by 5-HT_2B_-G_q_-PLCβ-PKCε signaling by regulating TRPV1 function.
